# Secular trends in incidence, survival, and health status at diagnosis of dementia in Sweden, 2007–2022

**DOI:** 10.1186/s13195-025-01928-y

**Published:** 2025-12-07

**Authors:** Mozhu Ding, Stina Ek, Fen Yang, Fang Fang, Katharina Schmidt-Mende, Karin Modig

**Affiliations:** 1 Unit of Epidemiology, Institute of Environmental Medicine, Karolinska Institutet, Nobels väg 13, Stockholm, 17177 Sweden; 2 Unit of Integrative Epidemiology, Institute of Environmental Medicine, Karolinska Institutet, Stockholm, Sweden; 3 Academic Primary Health Care Centre, Stockholm Region, Stockholm, Sweden; 4 Division of Family Medicine and Primary Care, Department of Neurobiology, Care Sciences and Society, Karolinska Institutet, Huddinge, Sweden

**Keywords:** Dementia diagnosis, Time trends, Nationwide, Survival, Frailty, Comorbidity

## Abstract

**Background:**

Although past screening cohorts have suggested a decline in the risk of dementia, it is important to monitor the population-level incidence and survival of diagnosed dementia, to inform care utilization and public health policies. This study provides nationwide analyses on time trends in the incidence of dementia diagnosis in Sweden between 2007 and 2022, as well as 5-year survival after a dementia diagnosis.

**Methods:**

Data from the total Swedish population aged ≥ 61 years during the period 2007–2022 were used. Incident dementia diagnoses were identified from specialist care and dispensed anti-dementia drugs. The annual incidence rate of dementia diagnosis was calculated for the period 2007–2022. The proportion of individuals that survived 5 years after dementia diagnosis was compared across years of diagnosis. Health status at dementia diagnosis was assessed by calculating Charlson Comorbidity Index and Hospital Frailty Risk Score.

**Results:**

Annual incidence rate of dementia diagnosis decreased from early 2010s and onwards, particularly among older age groups of 80–89 and ≥ 90 years. Mean age at dementia diagnosis remained constant, i.e., 82.2 years during 2007–2009 and 82.2 years during 2019–2022. The proportion of individuals with frailty at diagnosis increased from 74.3% in 2007–2009 to 80.6% in 2019–2022 (odds ratio (OR) = 1.42, 95% confidence interval (CI): 1.39–1.46); the proportion of individuals with comorbidities also increased over the same period. The proportion that survived 5 years since dementia diagnosis remained constant at 33% during 2007–2017 but improved over time when accounting for comorbidity and frailty level at diagnosis.

**Conclusions:**

While the incidence of dementia diagnosis has declined from early 2010s and onwards, patients diagnosed today are on average frailer and more comorbid than those diagnosed 15 years ago, which partly explains the lack of improvement in dementia survival over time. Enhancing healthcare planning for people with dementia diagnosis and improving their survival is still highly relevant.

**Supplementary Information:**

The online version contains supplementary material available at 10.1186/s13195-025-01928-y.

## Background

Dementia is a global health problem and major contributor to morbidity and mortality in old age [1]. In times of population aging and projected growth in dementia cases [2], it is important to monitor the population-level incidence of, and survival after, dementia diagnosis and how it has developed over time. For policy makers, such data is crucial to evaluate and plan for care use. For the individual patients, receiving a timely and accurate diagnosis is essential to ensure high-quality care.

Evidence about temporal trends in dementia incidence stems mostly from local research cohorts screening for dementia and has generally suggested declining incidence trends [3–5]. For example, a study found a 30% decline in dementia incidence when comparing two screening cohorts sampled 15 years apart in central Stockholm, Sweden [5]. Such data implies that the population’s underlying risk of dementia may have declined. However, in the clinical setting, dementia is underdiagnosed to a high extent in Sweden and worldwide, with approximately 50% of patients being undiagnosed [6, 7]. It is less known on the national level how the incidence of receiving a diagnosis of dementia has evolved over time. On the one hand, the incidence of dementia diagnosis may present an upward trend due to better detection and diagnostics [8]. On the other hand, the incidence of diagnosis may have declined if the population risk has decreased. Trend analysis based on real-world diagnosis data is thus needed to complement findings from the screening cohorts.

Most high-income countries such as Sweden have a remarkably aging population, which is partly due to a better survival of acute and/or chronic diseases in old age. Innovations in the treatment of life-threatening diseases such as myocardial infarction and some cancers have led to a substantial decrease in mortality [9–11]. In contrast, there is a lack of disease-modifying medications for dementia. However, a better understanding of dementia pathology and available interventions that focus on independence and quality of life [12], together with increased awareness of comorbidity management [13, 14], may have contributed to improved dementia diagnostics and care. It is thus relevant to examine the temporal trends in dementia survival and put it in context with the survival of the general older population. However, such studies are scarce, particularly on a national level [15, 16]. 

Furthermore, past data indicated that dementia onset may have been postponed to a higher age [17, 18]. As a result, dementia patients diagnosed today may be frailer and more likely affected by comorbidities than those diagnosed a decade ago, which in turn affects their prognosis and survival. Accounting for age and health status at the time of dementia diagnosis will reveal how patient characteristics differ across birth cohorts and how changing health status at the time of diagnosis affects survival trends, which is so far understudied.

Sweden is an ideal setting to examine long-term trends in dementia diagnosis. Sweden is known for its unique resources on high-quality health registers that have complete coverage of specialist diagnosis and prescribed drugs for the total population. With such data, this study aims to investigate 1) time trends in the incidence of dementia diagnosis in the Swedish older population aged ≥ 61 years during 2007–2022, 2) time trends in age and health status at dementia diagnosis, and 3) time trends in survival after dementia diagnosis and if the pattern differs by health status at diagnosis.

## Methods

Data was derived from the Aging and Health cohort, which was developed through linkage of several Swedish nationwide registers. All individuals in Sweden born before 1960 were identified from the Total Population Register and followed until the end of 2022. Through a unique personal identification number, the Total Population Register is linked to individual-level data from the National Patient Register (NPR) (since 1987), the Cause of Death Register (since 1952), the Prescribed Drug Register (PDR) (since 2005), and the Longitudinal Integrated Database for Health Insurance and Labor Market Studies (LISA) (since 1990). Data on primary care is additionally available for the Stockholm Region, which includes all primary care visits from 2013 to 2022.

To investigate the temporal trends in the incidence of dementia diagnosis, we included all individuals aged ≥ 61 years without a history of dementia diagnosis on January 1 st of each calendar year during the period 2007–2022. Individuals included in each calendar year were followed for incident dementia diagnosis identified in the NPR or PDR, whichever came first, until December 31st. The study period for trend analysis started from year 2007 to allow for a wash-out period of 1.5 years in the PDR for which data was available from July 2005 onwards. To study trends in age and health status at dementia diagnosis, we included all individuals with a first-ever dementia diagnosis between January 1 st, 2007, and December 31 st, 2022, in total 325,368 individuals. They were also divided into four groups according to time of diagnosis: January 1 st, 2007 to December 31 st,2009; January 1 st, 2011 to December 31 st, 2014; January 1 st, 2015 to December 31 st, 2018; January 1 st, 2019 to December 31 st, 2022. To examine time trends in dementia survival, individuals with a first-ever dementia diagnosis between 2007 and 2017 were followed for five years from the time of the dementia diagnosis until death, migration, and end of the 5-year period.

This study was performed in accordance with the ethical standards as laid down in the 1964 Declaration of Helsinki and its later amendments. Ethical approval for this study was obtained from the Regional Ethics Committee in Stockholm (2011-136-31/5, 2021–02880).

### Diagnosis of dementia

Diagnosis of dementia was identified from the NPR or PDR. The NPR contains hospital discharge records from inpatient care at national level since 1987 and data on specialist outpatient care since 2001. Information retrieved from this register includes the dates and discharge diagnoses of each visit which were coded according to the International Classification of Diseases (ICD) system. ICD-10 codes F00, F01, F02, F03, F05.1, G30 were used to identify dementia diagnosis.

Beginning in July 2005, information on dispensed medications collected at pharmacy was available in the PDR at national level, and all prescriptions were coded using the Anatomical Therapeutic Chemical (ATC) system. The first dispense of any anti-dementia drugs (ATC code N06D) between 2007 and 2022 was retrieved from this register. Because primary care data was not available for the entire country in the current study, using anti-dementia drugs could serve as a proxy of additional dementia cases (particularly Alzheimer’s Disease) that were likely diagnosed in primary care but not captured by specialist care.

### Assessment of health status and demographic variables at dementia diagnosis

Health status was measured through Charlson Comorbidity Index (CCI) and Hospital Frailty Risk Score (HFRS). The calculations of CCI and HFRS were based on diagnoses identified in the NPR in the 5 years preceding dementia diagnosis. CCI was calculated following the methods developed by Ludvigsson et al., an adapted version of the CCI to be used in Swedish registers [19]. The corresponding ICD-10 codes used to identify diseases in the CCI are presented in Supplementary Table 1. The HFRS was calculated following the methods developed by Gilbert et al. which used 109 ICD-10 codes weighted and summed to create a frailty score (Supplementary Table 2) [20]. Because CCI and HFRS were based on diagnoses received before dementia diagnosis, dementia did not contribute to the calculation of these scores. Data on date of birth, sex, and the highest attained education were retrieved from the LISA register. Education was categorized according to years of formal schooling into lower than high school (≤ 9 years), high school (10–12 years), and university (≥ 13 years).

### Statistical analysis

First, for each calendar year, individuals free of dementia diagnosis on January 1 st were followed for incident dementia diagnosis, death, migration, or December 31st. Annual incidence rate of dementia diagnosis was calculated as the number of people with new dementia diagnosis during the year divided by number of person-years at risk. To make the incidence rates comparable over 2007–2022, age- and sex-standardization was calculated using the 2015 Swedish population as the reference, to account for differences in the age and sex structure of populations over time. Statistical significance in the time trends of incidence rate of dementia diagnosis was tested using Cox regression models with calendar year as a continuous exposure variable, adjusting for age, sex, and education. Overall (≥ 61 years) and age group (61–69, 70–79, 80–89, and ≥ 90 years) specific incidence were included in the main analyses, and sex specific incidence was included in the supplementary analysis. Moreover, as a sensitivity analysis, dementia diagnoses from primary care were additionally added for the Stockholm Region where primary care data was available from 2013 onwards.

Next, among all individuals with incident dementia diagnosis between 2007-2022, mean and standard deviation (SD) of age, CCI, and HFRS at the time of dementia diagnosis, as well as distribution of age groups, sex, and education attainment, were compared across four time periods of dementia diagnosis. Logistic regression models were used to estimate the odds ratio (OR) and 95% confidence interval (CI) for having a CCI > 0 or HFRS > 0 by four time periods, adjusting for age, sex, and education. Furthermore, CCI and HFRS were treated as categorial variables and compared across four time periods using multinomial logistic regression models adjusting for age, sex, and education. CCI was categorized into 0, 1–2, 3–4, and ≥ 5, and HFRS into 0, 1–4, 5–14, and ≥ 15.

Finally, to study time trends in dementia survival, the proportion of individuals that survived 5 years since their first dementia diagnosis was compared across years of dementia diagnosis; p for trend was calculated using logistic regression models with years of dementia diagnosis as a continuous exposure variable, adjusting for age, sex, and education. This analysis was done both in the total sample and by different CCI and HFSR categories. The 5-year survival period was chosen because the average remaining life expectancy was estimated to be 4.8 years by previous meta-analysis [21]. Moreover, to examine how dementia survival has developed relative to the survival of the general population, 5-year mortality rate was compared between those with and without an incident dementia during each calendar year between 2007 and 2017. People with an incident dementia diagnosis were followed from the date of diagnosis, and those without incident dementia diagnosis were followed from January 1 st of the same year, until date of death, migration, or end of the 5-year period. Hazard ratios (HRs) and 95% CI comparing mortality among people with and without dementia diagnosis were estimated using Cox regression models adjusting for age, sex, and education.

## Results

### Time trends in the incidence of dementia diagnosis

**Fig. 1:**
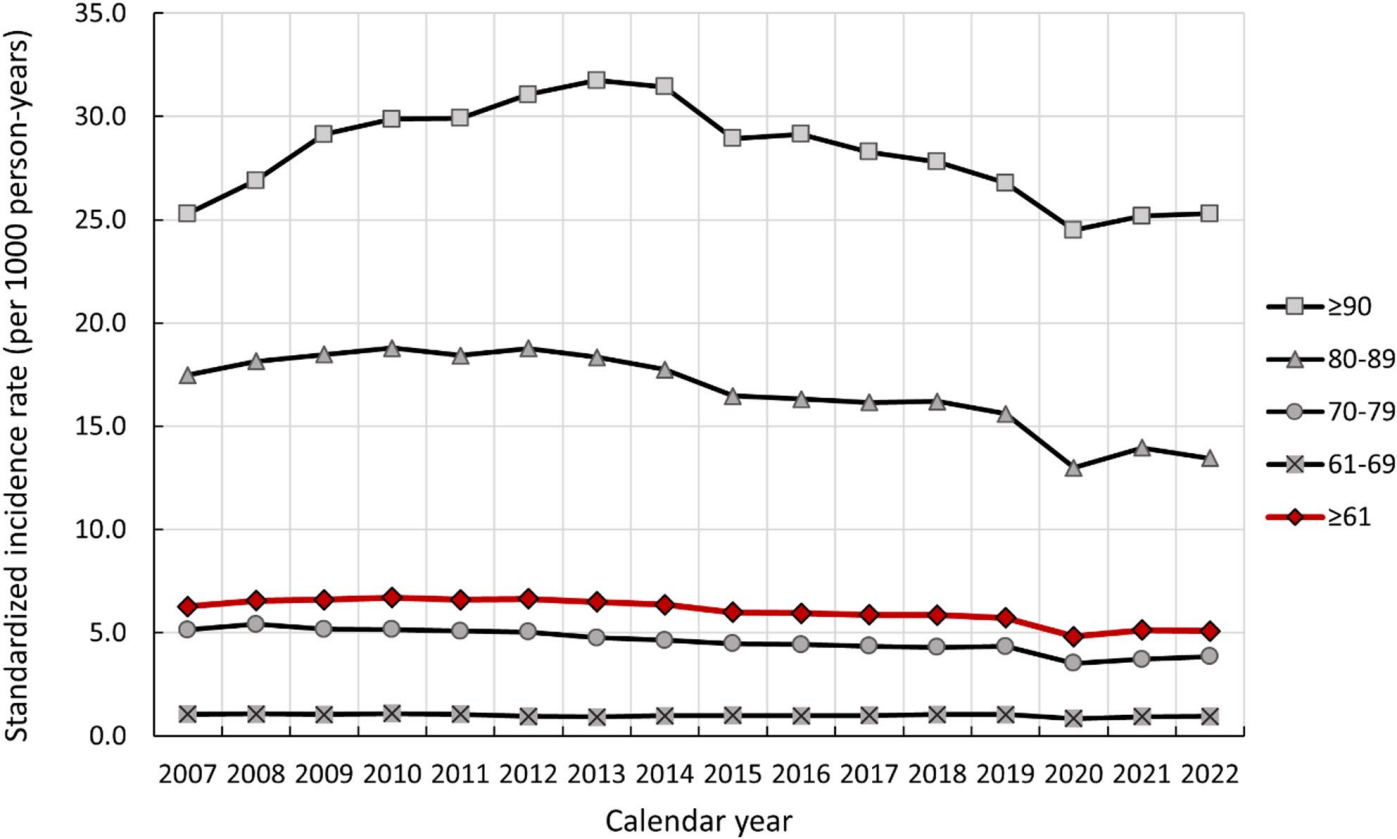
Time trends in annual incidence rate of dementia diagnosis per 1000 person-years in the total population and by age groups, 2007–2022. Incidence rate was age- and sex-standardized according to the 2015 Swedish population

Figure 1 shows time trends in the age- and sex-standardized annual incidence rate of dementia diagnosis between 2007-2022. The number of cases and population at risk for each calendar year are shown in Supplementary Table 3. In the total population aged ≥ 61 years, incidence rate of dementia diagnosis slightly increased from 6.27 cases per 1000 person-years in 2007 to 6.65 in 2012, after which it decreased to 5.08 in 2022 (*p* for trend < 0.001). Similar patterns were observed in age groups 70–79, 80–89, and ≥ 90 years, where the incidence rate decreased, with an annual decline of 2.0%, 2.6%, and 1.9% between 2012 and 2022, respectively (*p* for trend < 0.001 for each age group). In the age group 61–69 years, the incidence remained unchanged between 2007 and 2022 (p for trend = 0.704). Similar patterns were observed between men and women (Supplementary Fig. 1). Sensitivity analysis adding dementia diagnosis in primary care for Region Stockholm resulted in a higher incidence, but a similarly declining trend over time (Supplementary Fig. 2). The share of dementia diagnosis made in primary care alone increased with age, but within each age group it remained relatively stable over time (Supplementary Fig. 3).

### Time trends in age and health status at dementia diagnosis

Table 1 shows age and health status at the time of dementia diagnosis in four time periods. Mean age at dementia diagnosis did not change over time, i.e., 82.2 years during 2007–2009 and 82.2 years during 2019–2022. However, the distribution of age at diagnosis changed over time, where the share of patients aged ≥ 90 and 70–79 increased in more recent years, while the share of those aged 61–69 and 80–89 years decreased. Moreover, the share of women at dementia diagnosis decreased from 60.2% in 2007–2009 to 56.7% in 2019–2022, and so did the share of those with education lower than high school.

**Table 1:** Age and health status at the time of dementia diagnosis across four time periods

	2007–2009	2011–2014	2015–2018	2019–2022
N (%)	75,047 (23.1)	80,761 (24.8)	83,968 (25.8)	85,592 (26.3)
Age, mean (SD)	82.2 (7.3)	82.5 (7.5)	82.3 (7.6)	82.3 (7.5)
Age groups, n (%)				
61–69 years	5,001 (6.7)	5,510 (6.8)	5,595 (6.7)	5,087 (5.9)
70–79 years	21,295 (28.4)	22,139 (27.4)	25,110 (29.9)	27,187 (31.8)
80–89 years	39,003 (51.9)	40,303 (49.9)	39,693 (47.3)	39,765 (46.5)
≥ 90 years	9,748 (13.0)	12,809 (15.9)	13,570 (16.2)	13,553 (15.8)
Women, n (%)	45,202 (60.2)	48,161 (59.6)	48,359 (57.6)	48,554 (56.7)
Education*, n (%)				
Lower than high school	40,546 (54.0)	41,482 (51.4)	38,042 (45.3)	33,067 (38.6)
High school	21,235 (28.0)	26,162 (32.3)	29,841 (35.5)	33,176 (38.8)
University	8,610 (11.5)	11,341 (14.0)	14,543 (17.3)	18,040 (21.1)
Hospital Frailty Risk score (HFRS)				
Mean (SD)	3.2 (3.8)	4.7 (4.8)	5.2 (5.4)	5.5 (5.8)
HFRS > 0, n (%)	55,776 (74.3)	63,835 (79.0)	67,390 (80.3)	69,019 (80.6)
OR (95% CI) of HFRS > 0	Ref (1.00)	1.29 (1.26–1.32)	1.39 (1.36-0.42.36.42)	1.42 (1.39–1.46)
Charlson Comorbidity Index (CCI)				
Mean (SD)	1.0 (1.5)	1.3 (1.7)	1.4 (1.7)	1.4 (1.8)
CCI > 0, n (%)	39,534 (52.7)	44,617 (55.3)	46,215 (55.0)	46,112 (53.9)
OR (95% CI) of CCI > 0	Ref (1.00)	1.09 (1.07–1.12)	1.08 (1.06–1.11)	1.03 (1.01–1.06)

Odds ratios are adjusted for age at diagnosis, sex, and education

*Missing in education accounts for 2.9%

**Fig. 2:**
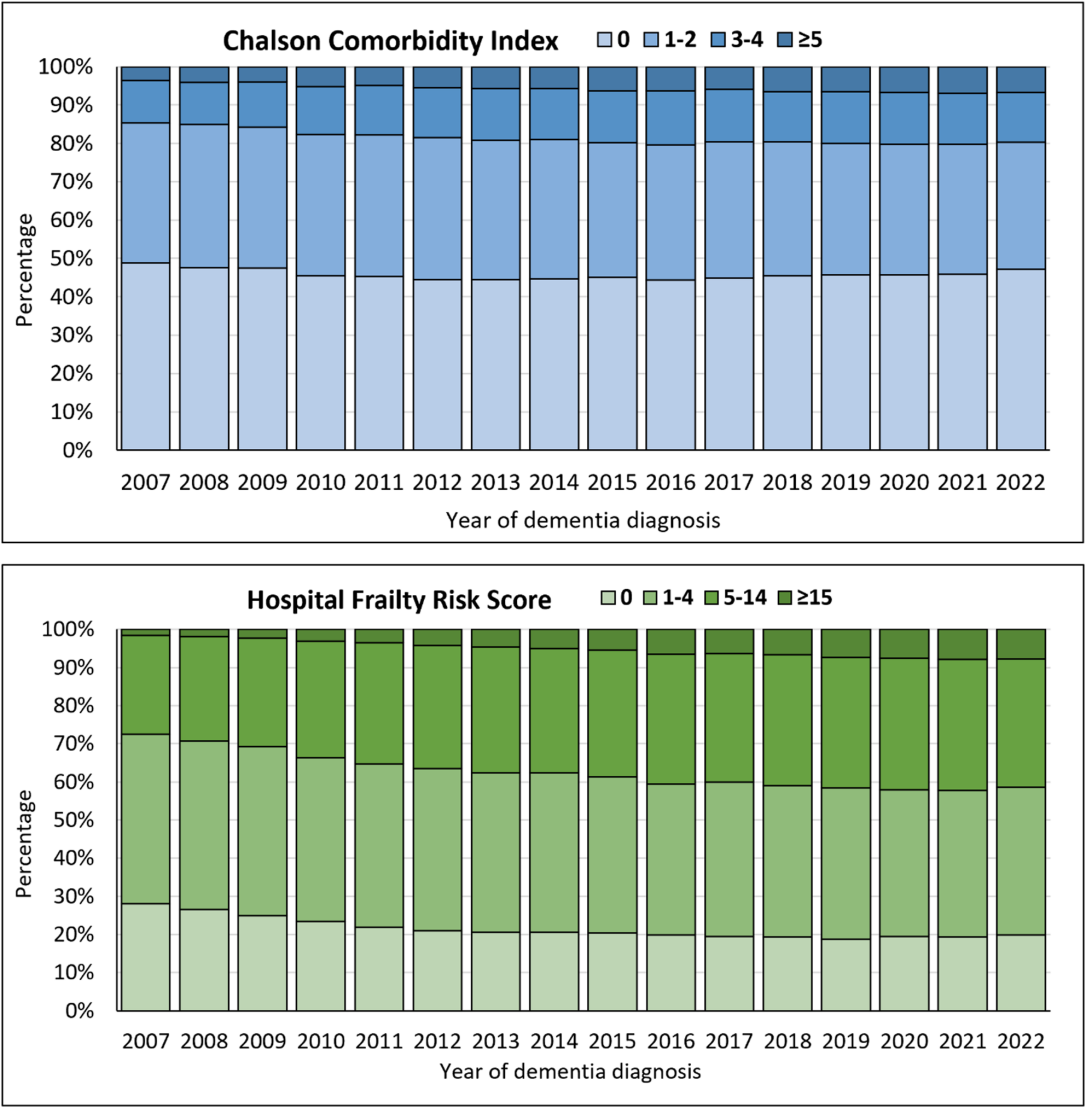
Time trends in the distribution of Charlson Comorbidity Index and Hospital Frailty Risk score categories at the time of dementia diagnosis, stratified by year of diagnosis

Frailty level at the time of dementia diagnosis increased from 2007-2009 to 2015–2018 and remained stable thereafter (Table 1). Specifically, 74.3% of the individuals had a HFRS > 0 at diagnosis in 2007–2009 while 80.6% had a HFRS > 0 at diagnosis in 2019–2022 (OR = 1.42, 95% CI: 1.39–1.46). The comorbidity level at dementia diagnosis only marginally increased, with 52.7% of individuals having a CCI > 0 in 2007–2009 and 53.9% in 2019–2022 (OR = 1.03, 95% CI: 1.01–1.06). Figure 2 shows the time trends in the distribution of CCI and HFRS categories by each year of dementia diagnosis. Overall, the share of very frail individuals (HFRS ≥ 15) at dementia diagnosis increased during 2007–2022, and so did the share of those with very high comorbidity level (CCI ≥ 5). Multinomial logistic regressions further showed that the proportion of CCI categories 1–2, 3–4, ≥ 5 and HFRS categories 1–4, 5–14, ≥ 15 were statistically significantly higher in more recent years (Supplementary Table 4).

### Time trends in survival after dementia diagnosis

**Fig. 3:**
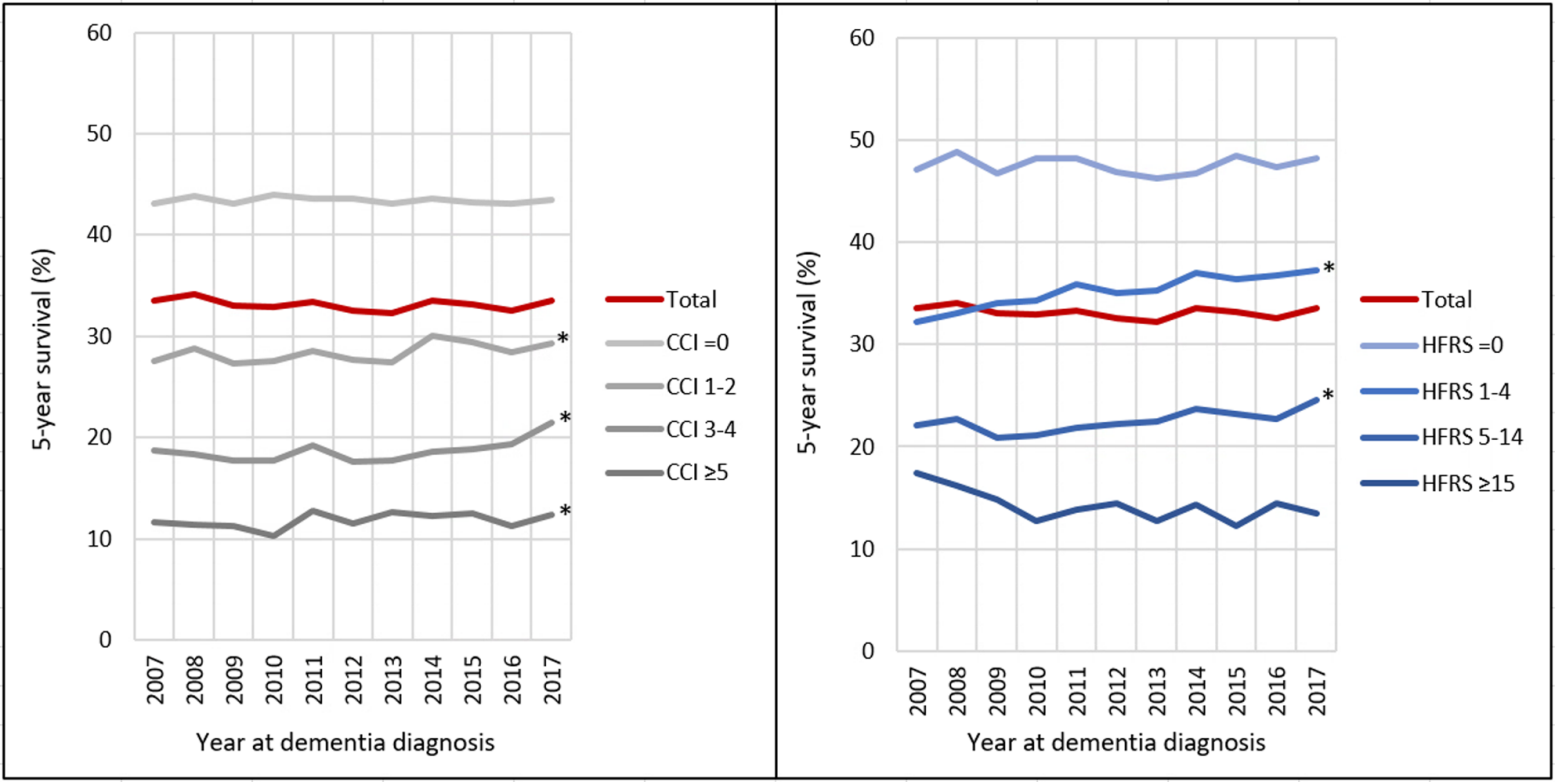
Time trends in the proportion alive within 5 years since a dementia diagnosis, stratified by CCI and frailty categories at the time of diagnosis. *p for trend < 0.05, adjusting for age at diagnosis, sex, and education

**Fig. 4:**
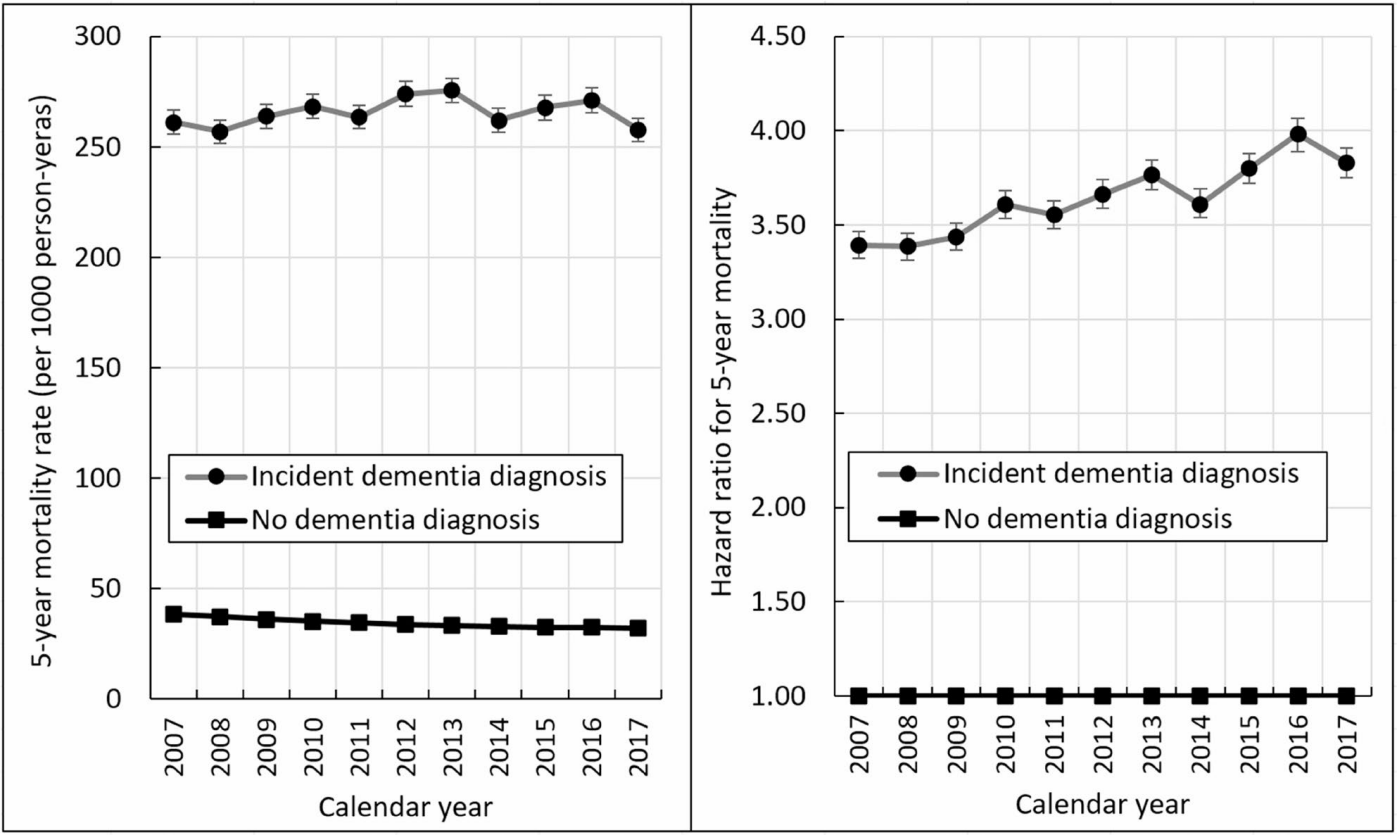
5-year mortality rate (left) and hazard ratio for 5-year mortality (right) comparing people with or without an incident dementia diagnosis by calendar year. Hazard ratios were adjusted for age, sex, and education

The proportion of individuals that survived 5 years after a dementia diagnosis did not change over time, i.e., 33.5% for 2007 and 33.6% for 2017 (Fig. 3). However, in the stratified analysis by health status at the time of dementia diagnosis, there was a significant improvement in 5-year survival among those with a CCI 1–2, 3–4, or ≥ 5 at diagnosis and those with a HFRS of 1–4 and 5–14 at diagnosis (p for trend < 0.05). Five-year survival among those with a CCI or HFRS of 0 or a HFRS of ≥ 15 at dementia diagnosis remained relatively stable. However, the 5-year mortality disadvantage among people with a dementia diagnosis compared to those without a dementia diagnosis widened from 2007 to 2017 (HR = 3.83, 95% CI: 3.75–3.91 in 2017; HR = 3.39, 95% CI: 3.32–3.47 in 2007), even though the absolute mortality rate among people with a dementia diagnosis did not vary substantially during the same period (Fig. 4). The number of deaths within 5 years among those with and without incident dementia diagnosis during each calendar year between 2007-2017 are shown in Supplementary Table 5.

## Discussion

In this nationwide study, we found that the incidence of dementia diagnosis increased slightly until 2012 after which it began to decline. Over the same period, the average age at diagnosis remained stable, whereas patients were increasingly frail and comorbid at the time of diagnosis. We further showed that the lack of overall survival improvement in people diagnosed with dementia was largely explained by increasing levels of frailty within this population. After accounting for frailty, survival improved over time. However, the mortality improvement for people with dementia diagnosis was smaller compared to their peers without dementia, resulting in a widening mortality gap between them.

Studies from screening cohorts in high-income countries such as Sweden [5], UK [22], US [3, 23], and France [24] have consistently reported a decline in dementia incidence from 1980 s and onwards, indicating a decrease in the population risk of dementia. On the other hand, our results suggest that such lowered risk did not manifest itself into a decline in the incidence of a clinical diagnosis until early 2010s. Before 2010 s, there was a slight increase in the incidence of dementia diagnosis, particularly in the two older age groups (80–89 and ≥ 90 years). This is in line with three other studies that reported a similar increase in dementia diagnosis incidence among older age groups during 1992–2013 in the Netherlands [8], 1999–2010 in Wales [25], and 1987–2013 in Sweden [26]. Although the diagnostic criteria of dementia have not changed substantially over the last decades, patient and physician’s awareness and perspectives of dementia may have changed and thus inflated the incidence of dementia diagnosis over time. After early 2010 s, it’s likely that the improvement in diagnosing people with dementia plateaued, and the decline in the incidence of dementia diagnosis was mostly driven by a lowered population risk. Moreover, the dip in dementia diagnosis during 2020 is most likely due to reduced care utilization in the covid-19 pandemic. However, it remains to be seen if the adoption of ICD-11 in Swedish health care in 2028 would alter the current decreasing trend, as ICD-11 introduces broader diagnostic criteria for dementia where memory impairment is no longer mandatory for diagnosis [27]. 

Our study is the first to show a difference over time in socio-demographics and health status at dementia diagnosis on a national level. The fact that we found no change in the mean age at dementia diagnosis during 2007–2022 may suggest a contribution of both declining incident dementia cases and earlier and timelier diagnosis in more recent years, given that local screening cohorts in both Sweden and other European countries have suggested postponement in dementia onset to higher ages [17, 28]. Studies using clinical samples have also reported higher Mini-Mental State of Examination scores among later-born individuals with dementia at the time of dementia diagnosis, suggesting a trend towards diagnosis in earlier stages of dementia [29]. We also found that the share of the oldest age group (≥ 90 years) at dementia diagnosis was higher in more recent years. Similarly, a recent study projected that the largest increase in the number of people with dementia in Wales and England has occurred, and will continue to occur, among the oldest age groups by 2040 [30]. This is possibly a result of better survival of age-related conditions (i.e., cardiovascular diseases and cancer), leading to a higher share of dementia patients that are old and frail. Indeed, in our study not only did the average frailty and comorbidity level at dementia diagnosis increased over time, but also the share of patients with very high disease burden (CCI ≥ 5 or HFRS ≥ 15). However, it may also reflect improved diagnostic procedures or changes in comorbidity documentation in the registers rather than worsening health status alone. Nevertheless, increasing disease diagnosis among later-born dementia patients may present particular challenges for health and social care system as dementia adversely affects the prognosis of co-existing conditions and is a key factor in care utilization [13, 14]. 

Past studies investigating time trends in dementia survival are relatively scarce. A decrease in dementia mortality has been reported by a local cohort in France between 1990s and 2000s [31], a nationwide study from Denmark between 1995-2015 [15], and the US Framingham Heart study between 1970s and 2000s [17]. In our study, the proportion of individuals that survived 5 years since their dementia diagnosis remained constant at 33% during 2007–2017. However, when stratifying the analysis by CCI and HFRS categories at the time of diagnosis, survival significantly improved among people with CCI >0 or HFRS 1–14. Such findings have two implications. First, the lack of improvement in dementia survival on the average level is at least partly driven by worsening health status at dementia diagnosis over time. Second, it is possible that better management of co-existing conditions among people with dementia has led to better survival in some disease subgroups. We nonetheless observed a widened gap in mortality risk between those with and without dementia diagnosis, suggesting increasing excess mortality due to dementia over time. These findings, together with worsening health status at dementia diagnosis, warrants enhanced initiatives to improve health and survival of current and future individuals with dementia.

This study has limitations. First, we did not have access to nationwide data on dementia diagnosed in primary care alone without use of anti-dementia drugs, leading to some missed cases. Our sensitivity analysis adding primary care dementia diagnosis showed a similar declining trend in incidence between 2013-2022 in the Stockholm Region, and the share of dementia diagnosis diagnosed in primary care alone remained relatively stable over time. However, it is not known to what extent this is generalizable at the national level. The proportion of missed cases could be higher in rural areas, as well as in later years when dementia investigation and diagnosis are increasingly adopted in primary care. It has been reported that people with dementia who were diagnosed in primary care alone were older and frailer than those diagnosed in specialist care [32]. Thus, the incidence of dementia diagnosis in our study may have been slightly underestimated in more recent years, particularly among the older age groups and in rural areas. Likewise, older and frailer dementia patients could also be more likely to consult primary care for their co-existing conditions, leading to an underestimation of comorbidity and frailty level among patients in more recent years (using data from specialized care alone). Moreover, as current anti-dementia drugs are mostly used to treat Alzheimer’s Disease and mixed dementia, other dementia types (e.g., vascular dementia) might be missed by ascertainment through dispensed anti-dementia drugs.

## Conclusions

In Sweden, the incidence of dementia diagnosis gradually declined from early 2010s and onwards, particularly among the oldest age groups. The declining incidence has been accompanied by increasing frailty levels and more comorbidities among patients. This changing health status at diagnosis over time has, at least partly, contributed to a lack of overall survival improvements after a dementia diagnosis, although survival improved in all but the frailest subgroup. Nevertheless, when compared to the rest of the population, the relative mortality disadvantage has increased. The results underscore the need for continued efforts to ensure timely and equitable dementia diagnosis, improved management of comorbidities and frailty in dementia patients, and the development of effective interventions to narrow survival inequalities.

## Supplementary Information

Supplementary Material 1.

## Data Availability

The datasets analyzed during the current study are not publicly available due to the General Data Protection Regulation in Sweden. Access to the data and the codes for data analyses can be permitted to external researchers after ethical vetting and establishment of a collaboration agreement. Contact the corresponding author for questions about data sharing (MD).

## Abbreviations

NPR: National Patient Register
PDR: Prescribed Drug Register
LISA: Longitudinal Integrated Database for Health Insurance and Labor Market Studies
ICD: International Classification of Diseases
ATC: Anatomical Therapeutic Chemical
CCI: Charlson Comorbidity Index
HFRS: Hospital Frailty Risk Score
SD: Standard deviation
OR: Odds ratio
CI: Confidence interval
