# Supplementary material for: Secular trends in incidence, survival, and health status at diagnosis of dementia in Sweden, 2007–2022
Source: Alzheimers Res Ther. 2025 Dec 7;17:260. doi: 10.1186/s13195-025-01928-y (PMC12696926; doi:10.1186/s13195-025-01928-y)
Supplement: Supplementary file 1 — Supplementary Material 1. [file 13195_2025_1928_MOESM1_ESM.docx]

**Supplementary Table 1.** Swedish ICD codes used to create the Charlson Comorbidity Index

| ICD 10 | Disease | Score |
| --- | --- | --- |
| I22-I23, I252 | Myocardial infarction | 1 |
| I11, I13, I255, I42–43, I50, I517 | Congestive heart failure | 1 |
| I70–73, I770-I771, K551, K558–559, R02, Z958–959 | Peripheral vascular disease | 1 |
| G45–46, I60–69 | Cerebrovascular disease | 1 |
| A810, F00–03, F051, G30–31 | Dementia | 1 |
| I26–27, J40-J47, J60–67, J684, J701, J703 | Chronic pulmonary disease | 1 |
| M05–06, M09, M120, M315, M32-M36 | Rheumatic disease | 1 |
| B18, I85, I864, I982, K70–71, K721, K729, K76, R162, Z944 | Liver disease | 2 |
| E10–14 | Diabetes mellitus | 2 |
| G114, G81–83 | Hemiplegia/paraplegia | 2 |
| I12–13, N01, N03, N05, N07, N08, N171, N172, N18, N19, N25, Z49, Z940, Z992 | Renal disease | 2 |
| C00–26, C30–34, C37–41, C43, C45–58, C60–76, C80–85, C88, C90–97 | Malignancy | 2 |
| C77–79 | Metastatic tumours | 6 |
| B20–24 | AIDS/HIV | 6 |

**Supplementary Table 2.** Swedish ICD 10 codes used to create the Hospital Frailty Risk Score

| **ICD10** | **ICD description** | **Weight** |
| --- | --- | --- |
| F00 | Dementia in Alzheimer’s disease | 7.1 |
| G81 | Hemiplegia | 4.4 |
| G30 | Alzheimer’s disease | 4.0 |
| I69 | Sequelae of cerebrovascular disease | 3.7 |
| R29 | Other symptoms and signs involving the nervous and musculoskeletal systems | 3.6 |
| N39 | Other disorders of urinary system (includes urinary tract infection and urinary incontinence) | 3.2 |
| F05 | Delirium, not induced by alcohol and other psychoactive substances | 3.2 |
| W19 | Unspecified fall | 3.2 |
| S00 | Superficial injury of head | 3.2 |
| R31 | Unspecified haematuria | 3.0 |
| B96 | Other bacterial agents as the cause of diseases classified to other chapters | 2.9 |
| R41 | Other symptoms and signs involving cognitive functions and awareness | 2.7 |
| R26 | Abnormalities of gait and mobility | 2.6 |
| I67 | Other cerebrovascular diseases | 2.6 |
| R56 | Convulsions, not elsewhere classified | 2.6 |
| R40 | Somnolence, stupor and coma | 2.5 |
| T83 | Complications of genitourinary prosthetic devices, implants and grafts | 2.4 |
| S06 | Intracranial injury | 2.4 |
| S42 | Fracture of shoulder and upper arm | 2.3 |
| E87 | Other disorders of fluid, electrolyte and acid-base balance | 2.3 |
| M25 | Other joint disorders, not elsewhere classified | 2.3 |
| E86 | Volume depletion | 2.3 |
| R54 | Senility | 2.2 |
| Z51 | Care involving use of rehabilitation procedures | 2.1 |
| F03 | Unspecified dementia | 2.1 |
| W18 | Other fall on same level | 2.1 |
| Z75 | Problems related to medical facilities and other health care | 2.0 |
| F01 | Vascular dementia | 2.0 |
| S80 | Superficial injury of lower leg | 2.0 |
| L03 | Cellulitis | 2.0 |
| H54 | Blindness and low vision | 1.9 |
| E53 | Deficiency of other B group vitamins | 1.9 |
| Z60 | Problems related to social environment | 1.8 |
| G20 | Parkinson’s disease | 1.8 |
| R55 | Syncope and collapse | 1.8 |
| S22 | Fracture of rib(s), sternum and thoracic spine | 1.8 |
| K59 | Other functional intestinal disorders | 1.8 |
| N17 | Acute renal failure | 1.8 |
| L89 | Decubitus ulcer | 1.7 |
| Z22 | Carrier of infectious disease | 1.7 |
| B95 | Streptococcus and staphylococcus as the cause of diseases classified to other chapters | 1.7 |
| L97 | Ulcer of lower limb, not elsewhere classified | 1.6 |
| R44 | Other symptoms and signs involving general sensations and perceptions | 1.6 |
| K26 | Duodenal ulcer | 1.6 |
| I95 | Hypotension | 1.6 |
| N19 | Unspecified renal failure | 1.6 |
| A41.9 | Other septicaemia | 1.6 |
| Z87 | Personal history of other diseases and conditions | 1.5 |
| J96 | Respiratory failure, not elsewhere classified | 1.5 |
| X59 | Exposure to unspecified factor | 1.5 |
| M19 | Other arthrosis | 1.5 |
| G40 | Epilepsy | 1.5 |
| M81 | Osteoporosis without pathological fracture | 1.4 |
| S72 | Fracture of femur | 1.4 |
| S32 | Fracture of lumbar spine and pelvis | 1.4 |
| E16 | Other disorders of pancreatic internal secretion | 1.4 |
| R94 | Abnormal results of function studies | 1.4 |
| N18 | Chronic renal failure | 1.4 |
| R33 | Retention of urine | 1.3 |
| R69 | Unknown and unspecified causes of morbidity | 1.3 |
| N28 | Other disorders of kidney and ureter, not elsewhere classified | 1.3 |
| R32 | Unspecified urinary incontinence | 1.2 |
| G31 | Other degenerative diseases of nervous system, not elsewhere classified | 1.2 |
| Y95 | Nosocomial condition | 1.2 |
| S09 | Other and unspecified injuries of head | 1.2 |
| R45 | Symptoms and signs involving emotional state | 1.2 |
| G45 | Transient cerebral ischaemic attacks and related syndromes | 1.2 |
| Z74 | Problems related to care-provider dependency | 1.1 |
| M79 | Other soft tissue disorders, not elsewhere classified | 1.1 |
| W06 | Fall involving bed | 1.1 |
| S01 | Open wound of head | 1.1 |
| A04 | Other bacterial intestinal infections | 1.1 |
| A09 | Diarrhoea and gastroenteritis of presumed infectious origin | 1.1 |
| J18 | Pneumonia, organism unspecified | 1.1 |
| J69 | Pneumonitis due to solids and liquids | 1.0 |
| R47 | Speech disturbances, not elsewhere classified | 1.0 |
| E55 | Vitamin D deficiency | 1.0 |
| Z93 | Artificial opening status | 1.0 |
| R02 | Gangrene, not elsewhere classified | 1.0 |
| R63 | Symptoms and signs concerning food and fluid intake | 0.9 |
| H91 | Other hearing loss | 0.9 |
| W10 | Fall on and from stairs and steps | 0.9 |
| W01 | Fall on same level from slipping, tripping and stumbling | 0.9 |
| E05 | Thyrotoxicosis [hyperthyroidism] | 0.9 |
| M41 | Scoliosis | 0.9 |
| R13 | Dysphagia | 0.8 |
| Z99 | Dependence on enabling machines and devices | 0.8 |
| U82.0 | Agent resistant to penicillin and related antibiotics | 0.8 |
| M80 | Osteoporosis with pathological fracture | 0.8 |
| K92 | Other diseases of digestive system | 0.8 |
| I63 | Cerebral Infarction | 0.8 |
| N20 | Calculus of kidney and ureter | 0.7 |
| F10 | Mental and behavioural disorders due to use of alcohol | 0.7 |
| Y84 | Other medical procedures as the cause of abnormal reaction of the patient | 0.7 |
| R00 | Abnormalities of heart beat | 0.7 |
| J22 | Unspecified acute lower respiratory infection | 0.7 |
| Z73 | Problems related to life-management difficulty | 0.6 |
| R79 | Other abnormal findings of blood chemistry | 0.6 |
| Z91 | Personal history of risk-factors, not elsewhere classified | 0.5 |
| S51 | Open wound of forearm | 0.5 |
| F32 | Depressive episode | 0.5 |
| M48.0 | Spinal stenosis (secondary code only) | 0.5 |
| E83 | Disorders of mineral metabolism | 0.4 |
| M15 | Polyarthrosis | 0.4 |
| D64 | Other anaemias | 0.4 |
| L08 | Other local infections of skin and subcutaneous tissue | 0.4 |
| R11 | Nausea and vomiting | 0.3 |
| K52 | Other noninfective gastroenteritis and colitis | 0.3 |
| R50 | Fever of unknown origin | 0.1 |

**Supplementary Table 3.** Number of incident dementia diagnosis and population at risk for each calendar year during 2007-2022 in the total population aged ≥61 years

| Calendar year | Population at risk | No. of incident dementia diagnosis |
| --- | --- | --- |
| **2007** | 1993788 | 18121 |
| **2008** | 2036960 | 18489 |
| **2009** | 2089649 | 18925 |
| **2010** | 2120954 | 19512 |
| **2011** | 2157923 | 19711 |
| **2012** | 2190908 | 20023 |
| **2013** | 2227105 | 20313 |
| **2014** | 2247399 | 20714 |
| **2015** | 2278316 | 20287 |
| **2016** | 2305623 | 20827 |
| **2017** | 2345359 | 21220 |
| **2018** | 2365891 | 21634 |
| **2019** | 2395268 | 21857 |
| **2020** | 2425249 | 19884 |
| **2021** | 2461708 | 21563 |
| **2022** | 2479766 | 22288 |

**
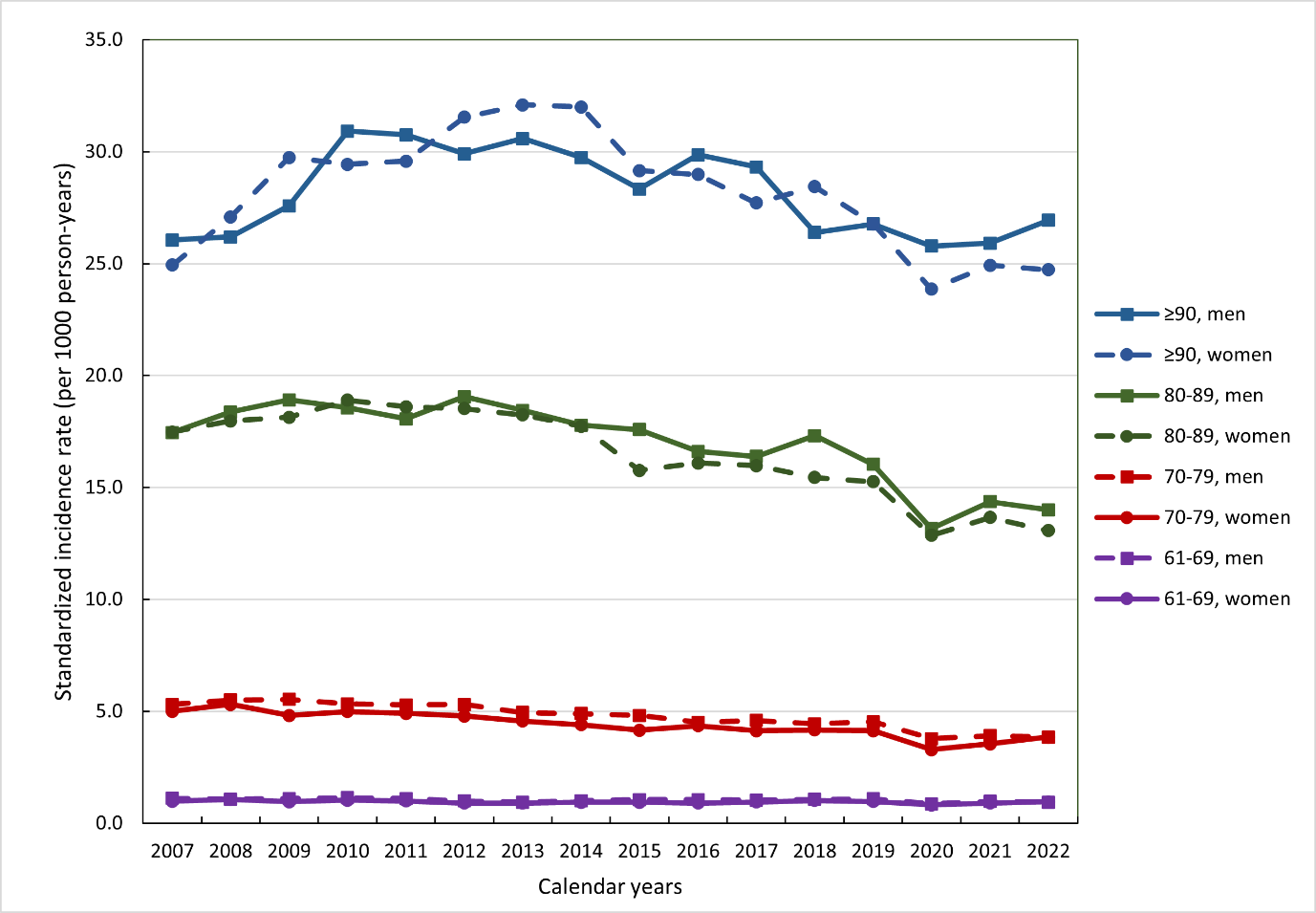
**

**Supplementary Figure 1**. Annual incidence rate of dementia diagnosis per 1000 person-years identified from specialist care and dispensed anti-dementia drugs, by sex and age groups during 2007-2022. Incidence rate was age-standardized according to the 2015 Swedish population.

**
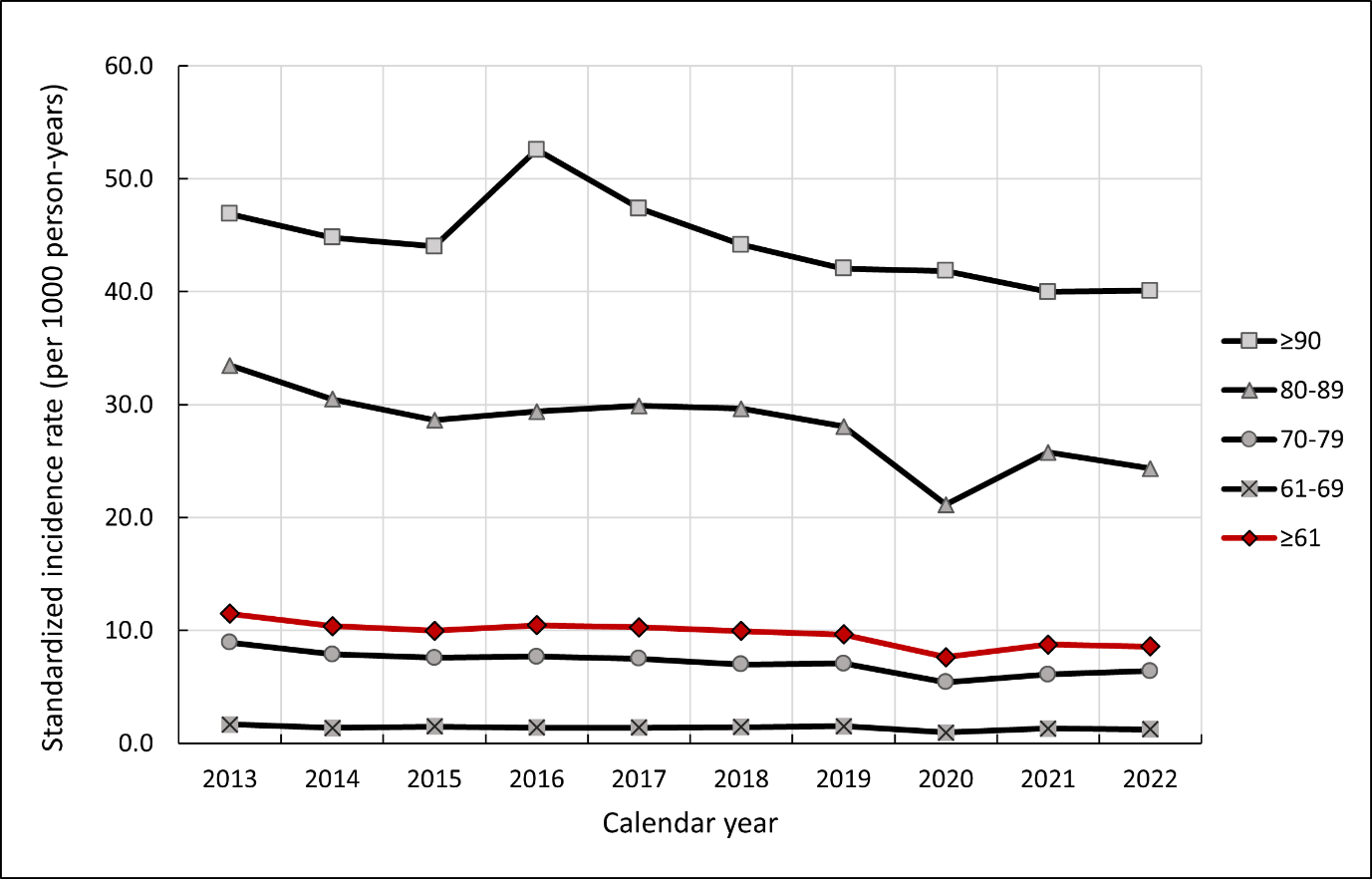
**

**Supplementary Figure 2**. Annual incidence rate of dementia diagnosis per 1000 person-years identified from specialist care, primary care, and dispensed anti-dementia drugs for Stockholm Region during 2013-2022.

**
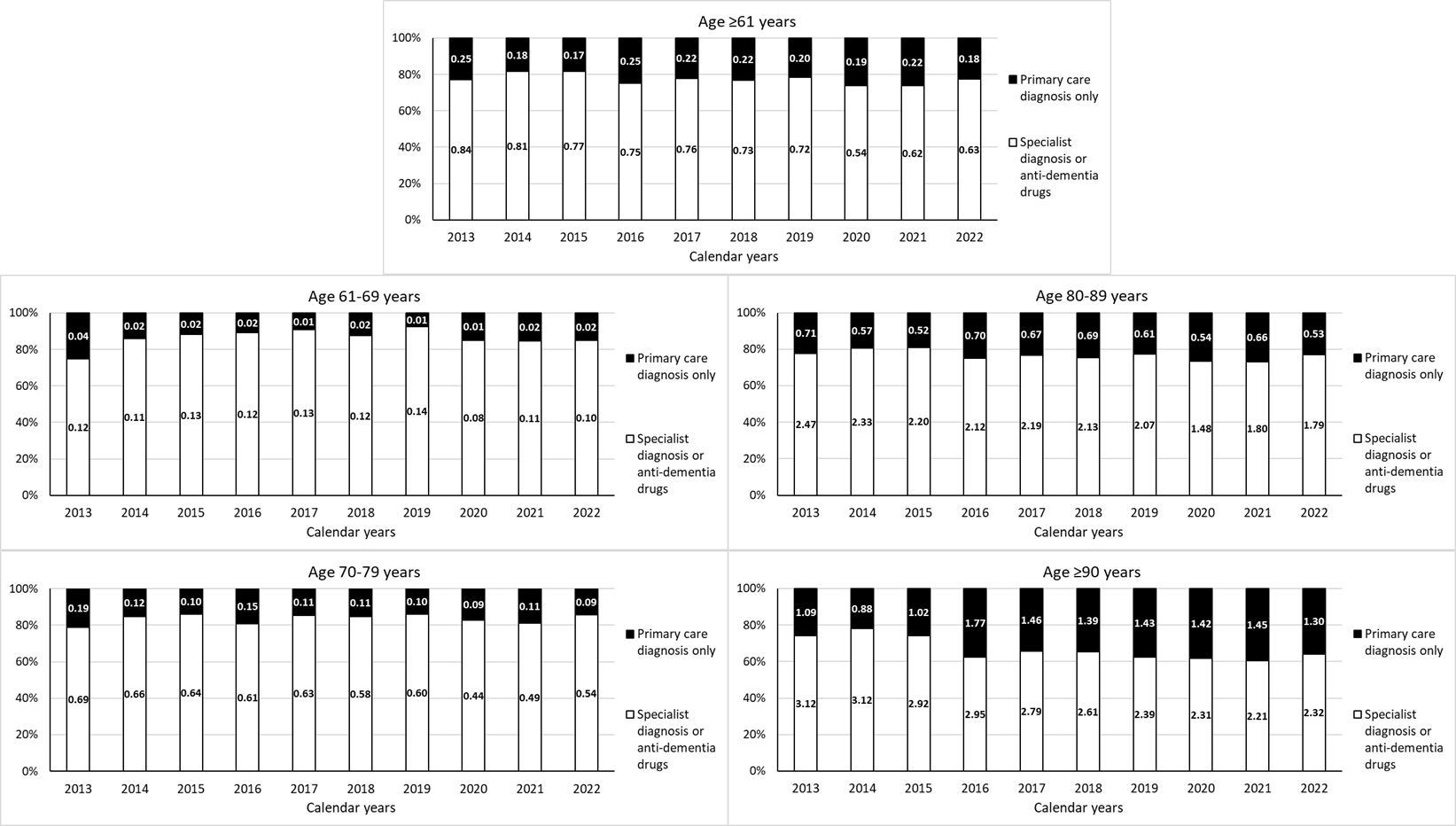
**

**Supplementary Figure 3.** Distribution of incident dementia diagnosis made in specialist care or dispensed anti-dementia drugs versus in primary care only, by age groups. The numbers in the bars represent the prevalence of dementia diagnosis.

**Supplementary Table 4**. Relative risk ratio and 95% confidence interval of Charlson Comorbidity Index and Hospital Frailty Risk Score categories compared to zero, by four time periods.

| **Time periods** | **Charlson Comorbidity Index categories vs 0** | | |
| --- | --- | --- | --- |
|  | 1-2 | 3-4 | ≥5 |
| 2007-2010 | Ref (1.00) | Ref (1.00) | Ref (1.00) |
| 2011-2014 | 1.04 (1.02-1.06)^a^ | 1.18 (1.15-1.22)^a^ | 1.34 (1.28-1.41)^a^ |
| 2015-2018 | 1.00 (0.97-1.02) | 1.21 (1.17-1.25)^a^ | 1.50 (1.44-1.57)^a^ |
| 2019-2022 | 0.93 (0.91-0.96)^a^ | 1.16 (1.13-1.20)^a^ | 1.57 (1.50-1.64)^a^ |
|  | **Hospital Frailty Risk Score categories vs 0** | | |
|  | 1-4 | 5-14 | ≥15 |
| 2007-2010 | Ref (1.00) | Ref (1.00) | Ref (1.00) |
| 2011-2014 | 1.18 (1.15-1.21)^a^ | 1.40 (1.36-1.44)^a^ | 2.39 (2.25-2.55)^a^ |
| 2015-2018 | 1.18 (1.15-1.21)^a^ | 1.56 (1.51-1.60)^a^ | 3.67 (3.46-3.90)^a^ |
| 2019-2022 | 1.16 (1.13-1.19)^a^ | 1.60 (1.56-1.64)^a^ | 4.56 (4.30-4.84)^a^ |

Relative risk ratios are from multinomial logistic regressions and adjusted for age at diagnosis, sex, and education. ^a^p<0.001

**Supplementary Table 5.** Number of deaths within five years among those with and without incident dementia diagnosis

| Calendar year | Individuals with incident dementia diagnosis | | Individuals without incident dementia diagnosis | |
| --- | --- | --- | --- | --- |
|  | No. of people | No. of deaths | No. of people | No. of deaths |
| **2007** | 18121 | 12042 | 1975667 | 346962 |
| **2008** | 18489 | 12184 | 2018471 | 345698 |
| **2009** | 18925 | 12676 | 2070724 | 344305 |
| **2010** | 19512 | 13095 | 2101442 | 341343 |
| **2011** | 19711 | 13141 | 2138212 | 340759 |
| **2012** | 20023 | 13505 | 2170885 | 340131 |
| **2013** | 20313 | 13765 | 2206792 | 340096 |
| **2014** | 20714 | 13771 | 2226685 | 340586 |
| **2015** | 20287 | 13563 | 2258029 | 339934 |
| **2016** | 20827 | 14042 | 2284796 | 345177 |
| **2017** | 21220 | 14097 | 2324139 | 346758 |
